# miR-762 activation confers acquired resistance to gefitinib in non-small cell lung cancer

**DOI:** 10.1186/s12885-019-6416-4

**Published:** 2019-12-10

**Authors:** Peng Ge, Lei Cao, Xin Chen, Ruijun Jing, Wanxia Yue

**Affiliations:** 1 Department of Cardiac & Thoracic Surgery, Second Affiliated Hospital of Xi’an Medical University, Xi’an, 710038 People’s Republic of China; 2 Department of Gynecology, Second Affiliated Hospital of Xi’an Medical University, Xi’an, 710038 People’s Republic of China; 3 Department of Pathology, Second Affiliated Hospital of Xi’an Medical University, No.167 Fangdong Avenue, Baqiao District, Xi’an, 710038 Shaanxi Province People’s Republic of China

**Keywords:** Non-small-cell lung cancer (NSCLC), Gefitinib resistance, miR-762, IL-6, ABR

## Abstract

**Background:**

Epidermal growth factor receptor (EGFR)-tyrosine kinase inhibitors (TKIs) (e.g. gefitinib) currently remain the first-line treatment for patients with advanced non-small-cell lung cancer (NSCLC) with activating EGFR mutation. However, acquired resistance to gefitinib, which occurs frequently through unidentified mechanisms, significantly attenuate therapeutic effectiveness. Previous miRNA microarray analysis reveals that expression levels of a conserved oncomiR miR-762 are significantly upregulated in gefitinib-resistant NSCLC cells. We therefore aim to elucidate the role and underlying mechanisms of miR-762 during the pathogenesis of gefitinib resistance.

**Methods:**

miR-762 expression in gefitinib-resistant NSCLC tissues and cells was evaluated using RT-qPCR. The potential regulation of miR-762 expression by IL-6 was studied using pharmacological and biochemical approaches. Effects of miR-762 manipulation on sensitivity to gefitinib was assessed using MTT, apoptotic ELISA and xenograft model. Finally, the posttranscriptional regulation of active BCR related protein (ABR) by miR-762 was determined using luciferase assay and site-directed mutagenesis.

**Results:**

miR-762 expression was upregulated in gefitinib-resistant NSCLC tissues and cells, and this upregulation predicted a poor post-chemotherapy prognosis in NSCLC patients. miR-762 upregulation, induced by IL-6 signaling, significantly enhanced cell survival and rendered NSCLC cells unresponsiveness to gefitinib-elicited cell death. We finally provided the evidence that the oncogenic effect of miR-762 was mediated mainly through posttranscriptional repression of ABR in gefitinib-resistant NSCLC cells.

**Conclusions:**

Our findings provide a rationale for future efforts testing miR-762 inhibition and ABR restoration co-treatment in patients with recurrent EGFR mutant NSCLC to therapeutically combat the heterogeneity of EGFR-TKIs resistance mechanisms.

## Background

Currently, lung cancer (LC) represents one of the most aggressive cancer types with highest mortality in both developing and developed countries. Given that the constitutive activation of epidermal growth factor receptor (EGFR) signaling pathway plays an essential role in the development and progression of advanced non-small cell lung cancer (NSCLC) whose tumors harbor EGFR-activating mutations [1], EGFR-tyrosine kinase inhibitors (TKIs) including gefitinib, erlotinib and afatinib, have become the standard of treatment for mutation-positive, advanced-stage non-squamous NSCLC [2]. Regardless of the certain improvement in clinical outcomes driven by EGFR-TKIs, however, frequent occurrence of acquired resistance to EGFR-TKIs significantly impedes therapeutic effectiveness and potentiates unfavorable prognosis in patients with EGFR-mutant NSCLC. Considering that neo-adjuvant chemoradiotherapy has limited therapeutic effects and current prediction of disease prognosis still depends on conventional pathologic variables (tumor grade and metastasis status), there is an unequivocal need for elucidating the molecular underpinnings of NSCLC to develop more potent diagnostic biomarkers and therapeutic strategies, especially during the pathogenesis of EGFR-TKIs resistance.

MicroRNAs (miRNAs), a class of small, well-conserved, non-coding RNAs regulating a vast array of cellular processes by targeting the three prime untranslated region (3′-UTR) of target gene, are emerging as the largest contributors to the diversity of cellular functions under both physiological and pathological conditions. Recent profiling and functional studies have demonstrated that a panel of miRNAs (miR-608, − 4513, − 497, −499a [3], −23a [4] and et al.) are aberrantly expressed in EGFR-TKIs-resistant NSCLC, and dysregulation of these miRNAs regulates fundamentally the pathogenesis of EGFR-TKIs resistance via modulation of multiple cancerous functions including deregulated cell proliferation/apoptosis, maintenance of cancer stem cells and activation of alternative signaling (HGF, Met, AXL and IGF-1R). Of particular interest, a very recent study using high-throughput analysis (miRNA microarray analysis) demonstrates that miR-762 is significantly induced in experimentally established gefitinib-resistant NSCLC cells. More importantly, silencing IGF-1R expression in gefitinib-resistant NSCLC cells totally abolishes miR-762 induction, suggesting a close connection between miR-762 expression and IGF-1R signaling pathway [5]. miR-762 has been shown to be a novel prognostic biomarker for muscle-invasive bladder cancer and ovarian cancer [6, 7]. Nevertheless, the function of miR-762 (either as oncomiR or tumor suppressor miRNA) and its corresponding mechanisms in gefitinib resistance remain poorly understood.

Acting as one of the most important EGFR bypass signaling pathways, IGF-1R signaling has been demonstrated to regulate essentially the EGFR-TKIs resistance in NSCLC [5, 8], the current study was designed to elucidate whether changes in miR-762 expression levels correlate to the development of resistance to gefitinib, and to further identify the putative targets of miR-762 using in vitro cell-based systems. Our profiling and functional data help us to propose that miR-762 acts as an oncomiR, whose expression is gradually upregulated along the development of gefitinib resistance, presenting a potential therapeutic vulnerability node for exploitation in the NSCLC treatment.

## Methods

### Cell treatment

Human NSCLC cell lines, including A549 (cat. No.: ATCC-CCL-185), NCI-H820 (cat. No.: ATCC-HTB-181), NCI-H2170 (cat. No.: ATCC-CRL-5928), NCI-H1650 (cat. No.: ATCC-CRL-5883), NCI-H1993 (cat. No.: ATCC-CRL-5909), NCI-H2126 (cat. No.: ATCC-CCL-256), NCI-H1975 (cat. No.: ATCC-CRL-5908), NCI-H1299 (cat. No.: ATCC-CRL-5803), NCI-H1648 (cat. No.: ATCC-CRL-5882), NCI-H1703 (cat. No.: ATCC-CRL-5889), NCI-H2347 (cat. No.: ATCC-CRL-5942) and a normal human lung epithelial cell line NuLi-1 (cat. No.: ATCC-CRL-4011) were obtained from ATCC (Manassas, VA, USA) during the year 2018. Another NSCLC cell line PC-9 (cat. No.: RRID-CVCL-B260) was obtained from the State Key Laboratory (SKL) of Oncology in South China during the year 2018. All cell lines were recently authenticated by STR profiles with ABI3500xl Genetic Analyzer, and all cells were recently verified no contamination with mycoplasma before experiments. Cells were cultured in RPMI1640 medium containing 10% fetal bovine serum (Thermo Fisher Scientific, Shanghai, China) at 37 °C in a 5%-CO_2_ incubator. Establishment of gefitinib-resistant NSCLC cells was achieved by incubating cells to gradually increasing concentrations of gefitinib (Sigma-Aldrich) for ~ 11 months (The maximum gefitinib concentrations were 5 and 40 μM for PC-9/GR and A549/GR, respectively). The gefitinib-resistant sublines PC-9/GR and A549/GR were finally maintained in medium containing 5 or 40 μM of gefitinib, respectively [9, 10]. To study the potential regulation of miR-762 by various proinflammatory cytokines, A549 cells were incubated with different cytokines, including IL-1α (5 ng/ml,), IL-1β (10 ng/ml,), IL-6 (10 ng/ml,) and IL-8 (50 ng/ml,), for 24 h. All these recombinant human cytokines were purchased from PeproTech (Suzhou, Jiangsu Province, China). To study the potential effects of IL-6 stimulation on miR-762 expression, cells were stimulated either with 10 ng/ml of IL-6 for different durations, or stimulated with different doses of IL-6 for 24 h. To investigate the STAT3-dependent regulation of miR-762 by IL-6, cells were stimulated with 10 ng/ml of IL-6 for 24 h, in the presence or absence or 5 μM of WP1066 (Selleck, Shanghai, China). To further validate the STAT3-dependent regulation of miR-762 by IL-6, A549 cells were transiently transfected with *STAT3* siRNA or Ctrl siRNA (Santa Cruz Biotechnology, Shanghai, China) using Lipofectamine® 2000 (Thermo Fisher Scientific) for 48 h. The specificity and effectiveness of the *STAT3* siRNA has been validated [11]. To manipulate the expression levels of miR-762, NSCLC cells were transfected for 48 h with miR-762 inhibitors/mimics, along with the corresponding negative controls (NC) (Thermo Fisher Scientific, Shanghai, China), using Lipofectamine®2000 [12, 13]. To generate the PC-9 or A549 cells stably expressing the exogenous active BCR related gene (ABR), cells were transfected with pCMV3-ABR or empty vector (Sinobiological, Beijing,China) for 48 h, followed by selection with 200 μg/ml of hygromycin (Thermo Fisher Scientific).

### Cytotoxicity upon gefitinib challenge

48 h after transfection, LC cells were seeded at the density of 0.4 × 10^4^ cells/well in a 96-well plate. Cells were then treated with different doses of gefitinib (8 μM for PC-9/GR, 60 μM for A549/GR, 0.2 μM for PC-9 and 12.5 μM for A549 cells) for 24 or 48 h. Cell viability and apoptosis were assayed using a MTT Assay Kit (Abcam, Shanghai, China) and the ApoStrand™ ELISA Apoptosis Detection Kit (ENZO LIFE, Farmingdale, NY, USA) at 590 and 405 nm, respectively. The relative cell viability (%) was expressed as a percentage of viable cell proportion for treated sample compared to that of mock control at 0 h.

### In vivo chemosensitivity

In vivo gefitinib sensitivity was evaluated using a xenograft model [3]. Briefly, LC cells were resuspended in culture medium and injected subcutaneously into the flanks of 6-week-old male BALB/c nude mice at the concentration of 1.0 × 10^6^ cells/200 μl of medium (*n* = 7/group). When xenografts grew to ~ 50 mm^3^, mice were administrated orally with gefitinib [10 mg/kg, dissolved in a volume of 150 μl of vehicle control (Tween 80-ethanol-H_2_O, 1:1:98)] on a daily basis. The control mice received the same volume of vehicle control. Tumor volume was measured once a week, using the formula: V (mm^3^) = length×width^2^ × 0.5. At the end of 32 days after cell inoculation, mice were euthanized by carbon dioxide inhalation followed by cervical dislocation. The BALB/c nude mice were obtained from animal facility in Second Affiliated Hospital of Xi’an Medical University. All procedures involved in the animal work were strictly conformed to the *Guide for the Care and Use of Laboratory Animals* from NIH, and were approved by IACUCs of Second Affiliated Hospital of Xi’an Medical University (#XAMU-2007-134-1A).

### Quantitative RT-PCR (RT-qPCR)

Total RNA was isolated and purified using the mirVana™ miRNA Isolation Kit (Thermo Fisher Scientific). Subsequent reverse transcription (RT) was conducted using the Applied Biosystems TaqMan MicroRNA Reverse Transcription Kit (Thermo Fisher Scientific). To detect miRNA expression, qPCR was conducted with the aid of the Applied Biosystems TaqMan MicroRNA Assays, using U6 expression for normalization. To detect other target transcripts, qPCR was performed using the SYBR Green Master Mix (Bio-Rad, Shanghai, China), as described earlier [14]. Amplification of *ACTIN* was served as the internal control. Relative expression levels were quantified using the the 2^−ΔCt^ method [15]. The primers used were: *ABR*, 5′-AGCCGAGATATGAGCCTGAA-3′ and 5′-CCTCGATACCCCTCTTCTCC-3′ [16]; *ACTIN*, 5′-AGCACAATGAAGATCAAGAT-3′ and 5′-TGTAACGCAACTAAGTCATA-3′ [3]; *IL-1A*, 5′-AACCAGTGCTGCTGAAGGA-3′ and 5′-TTCTTAGTGCCGTGAGTTTCC-3′; *IL-1B*, 5′-CTGTCCTGCGTGTTGAAAGA-3′ and 5′-TTGGGTAATTTTTGGGATCTACA-3′; *IL-6*, 5′-TTCAATGAGGAGACTTGCCTG-3′ and 5′-ACAACAACAATCTGAGGTGCC-3′ and *IL-8*, 5′-ACTCCAAACCTTTCCACCC-3′ and 5′-AAACTTCTCCACAACCTCTG-3′ [17].

### Immunoblotting

Total protein was prepared using the Total Protein Extraction Kit (Merck Limited, Hong Kong, China). Protein samples (~ 25 μg) were separated by SDS-PAGE and transferred to a PVDF membrane (Sigma-Aldrich). Membranes were then incubated with different primary antibodies (Additional file 4: Table S1) at 4 °C overnight, followed by incubation with an HRP-conjugated secondary antibody (Vector Laboratories, Burlingame, CA,USA) at rt. for 2 h. Final immunobands were visualized with the assistance of a Pierce SuperSignal Kit (Thermo Fisher Scientific).

### Luciferase reporter assay

We amplified the full-length 3’UTR of human *ABR* gene from cDNA synthesized from total RNA of A549 cells using a GeneRacer Kit (Thermo Fisher Scientific), and cloned it into pGL3-Basic Vector using In-Fusion® HD Cloning Kit (Takara, Beijing, China). The site-directed mutagenesis was achieved with the aid of the QuikChange II site-directed mutagenesis kit (Agilent, Beijing, China). For reporter assay, we co-transfected miR-762 mimics/Mimics-NC (25 pmol/well) and pGL3-ABR 3’UTR-Luc reporter (0.25 μg/well), together with 0.001 μg of the Renilla luciferase reporter (Promega, Beijing, China), into subconfluent proliferating NIH/3 T3 cells using Lipofectamine®2000. 24 h later, cells were harvested and the relative luciferase (Luc) activity was measured using the Dual-Luciferase® Reporter (DLR™) System from Promega.

### Human samples

Upon receipt of written informed consent from all participants, a total of 59 patients with recurrent EGFR mutant NSCLC who had been treated with epidermal growth factor receptor-tyrosine kinase inhibitors (EGFR-TKIs) were enrolled from Department of Cardiac & Thoracic Surgery, Second Affiliated Hospital of Xi’an Medical University during the period from 2008 to 2016. Resistance to EGFR-TKIs was defined based on the criteria described elsewhere [18]. Tumor samples were obtained during rebiopsy. The human study was conducted in accordance with the *Declaration of Helsinki* (2013), and all the protocols involved were approved by the ethics committee review board at Second Affiliated Hospital of Xi’an Medical University (#XAMU-2007-134-1B). The clinicopathologic features of the patients were presented in Table 1.

**Table 1:** Pre-treatment characteristics of 59 patients with EGFR mutant NSCLC treated with first line EGFR-TKIs

Clinical characteristics	Patient number (%)
Sex	
Male	36 (61.0)
Female	23 (39.0)
Age	
< 60	40 (67.8)
≥ 60	19 (32.2)
Smoking status	
Never smoked	17 (28.8)
Former smoker	28 (47.5)
Current smoker	14 (23.7)
ECOG*performance status	
0	21 (35.6)
1	32 (54.2)
2	4 (6.8)
3	2 (3.4)
Disease stage	
IIIB	18 (30.5)
IV	41 (69.5)
Brain metastasis	
-	51 (86.4)
+	8 (13.6)
Bone metastasis	
-	34 (57.6)
+	25 (42.4)
Type of EGFR mutation	
Exon 19 deletion	46 (78.0)
L858R	12 (20.3)
Other	1 (1.7)
EGFR-TKI type as first-line therapy	
Gefitinib	26 (44.1)
Erlotinib	31 (52.5)
Afatinib	2 (3.4)

*EGFR* epidermal growth factor receptor, *NSCLC* non-small cell lung carcinoma, *EGFR-TKIs* epidermal growth factor receptor tyrosine kinase inhibitors, *ECOG* Eastern Cooperative Oncology Group

### Data analysis

Data were analyzed using GraphPad Prism 5 from at least three independent experiments, and were expressed as mean ± SD. Statistical comparisons were determined by either *Student’s t* test or one-way ANOVA, followed by Tukey post-hoc analyses wherever appropriate. The association between miR-762 and *ABR* mRNA levels was assessed using the *Pearson Chi-Square* test and the survival difference was analyzed using Kaplan–Meier method. *P* < 0.05 was considered statistically significant.

## Results

### Upregulation of miR-762 is associated with gefitinib resistance in NSCLC cells

**Fig. 1:**
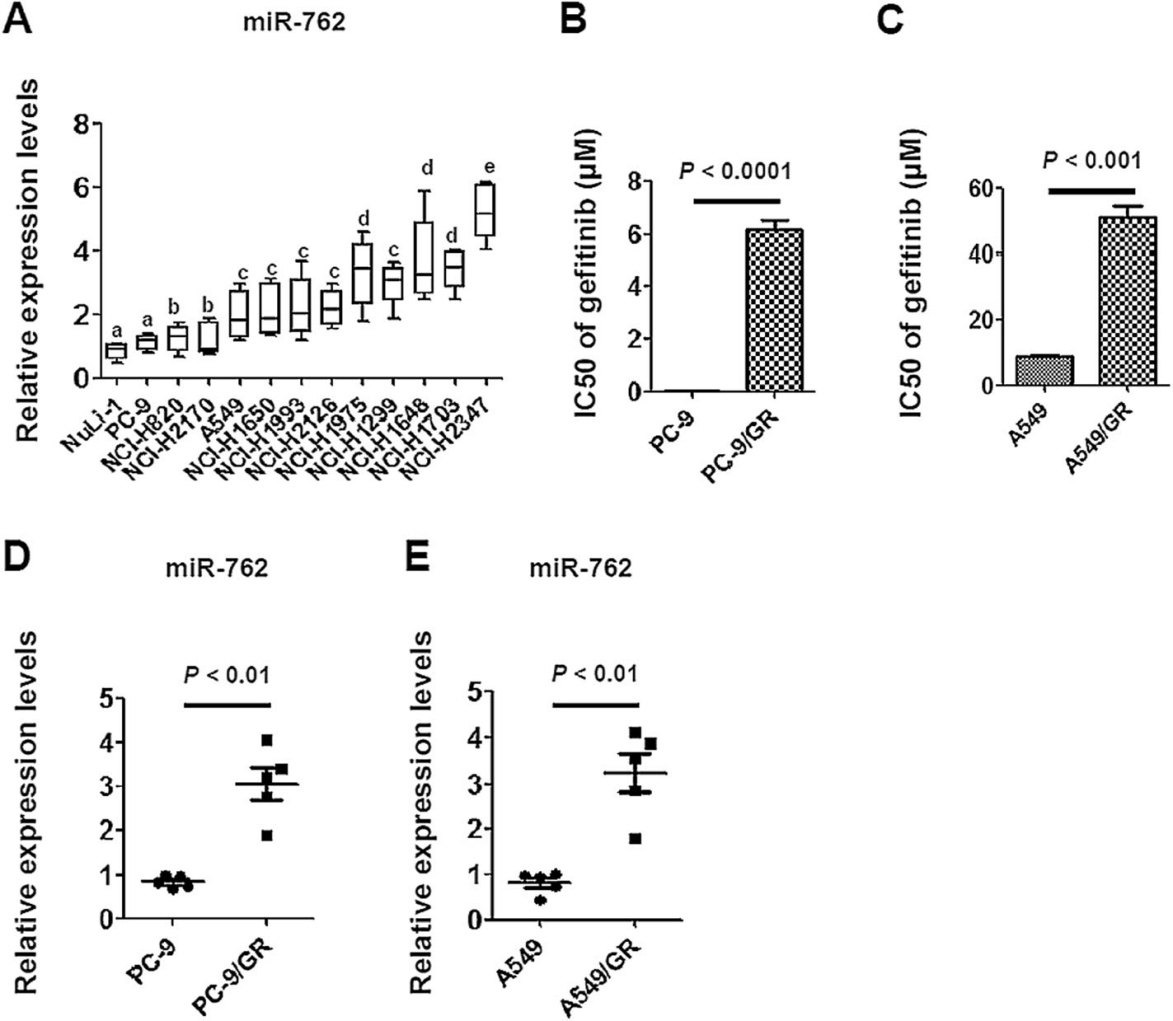
Elevated miR-762 expression is associated with gefitinib resistance in NSCLC cells. **a** RT-qPCR analysis of miR-762 expression in different NSCLC cells. Relative expression levels of miR-762 were obtained in each sample by normalization of the expressions of miR-762 to that of the U6 snRNA signal. For presentation of data, expression levels of miR-762 from NuLi-1 cells were taken as 100% and the others were normalized accordingly. Each value is a mean ± S.E.M. from three independent experiments. Different superscript letters denote groups that are statistically different (*P* < 0.05). **b** The IC50 value (inhibitory concentration to produce 50% cell death) following a 48-h exposure to gefitinib was determined in PC-9 and PC-9/GR cells using MTT assay. **c** The IC50 value following a 48-h exposure to gefitinib was determined in A549 and A549/GR cells using MTT assay. **d**-**e** Characterization of miR-762 expression in different NSCLC cells using RT-qPCR. The value indicates the relative expression levels of miR-762 in the cells (PC-9/PC-9/GR and A549/A549/GR cells) at different batches of gefitinib resistant induction

To study the potential involvement of miR-762 in the regulation of chemosensitivity, we evaluated the expression profile of miR-762 in a bunch of NSCLC cells. Based on the IC50 values calculated from the MTT assays, we categorized the NSCLC cells into three groups: most sensitive to gefitinib (PC-9, NCI-H820 and NCI-H2170, IC50ϕ5.0 μM), modestly sensitive to gefitinib (A549, NCI-H1650, NCI-H1993 and NCI-H2126, 5.0 μM ≦IC50≦25.0 μM) and resistant to gefitinib (NCI-H1975, NCI-H1299, NCI-H1648, NCI-H1703 and NCI-H2347, IC50≧25.0 μM) (Table 2). Subsequent RT-qPCR analyses revealed that miR-762 expression levels correlated negatively to the sensitivity to gefitinib in NSCLC cells, with the lowest values being observed in PC-9 cells and a normal human lung epithelial cell line (NuLi-1) and the highest values being detected in NCI-H2347 cells (Fig. 1a). We further chose PC-9 and A549 cells in our in vitro study, mainly based on three criteria: i) These two cell lines were shown to be relatively sensitive to gefitinib. ii) miR-762 expression is relatively low in these two cell lines. iii) These two cell lines are most commonly used NSCLC cells for the induction of gefitinib resistance [19]. Notably, miR-762 expression appeared to be irrelevant to the histological types in NSCLC cells (Fig. 1a, Table 2). To verify this observation, we generated the gefitinib-resistant sublines by exposing cells to gradually increasing concentrations of gefitinib. The resultant PC-9/GR and A549/GR cells exhibited more than 4.5-fold higher levels in gefitinib resistance, respectively (Fig. 1b and c). Interestingly, miR-762 expression was significantly stimulated in gefitinib-resistant cells compared to their parental cells (Fig. 1d and e). These findings suggest that miR-762 upregulation may be associated with the pathogenesis of gefitinib resistance in NSCLC cells.

**Table 2:** Sensitivity to gefitinib treatment for different NSCLC cells

Cell lines	Histology	EGFR mutation	IC50 of gefitinib (μM)
PC-9	AD	E746-A750 del	0.047 ± 0.012
NCI-H820	AD	E746-E749 del	2.783 ± 0.854
NCI-H2170	SQ	WT	4.254 ± 0.752
A549	AD	WT	9.172 ± 1.228
NCI-H1650	AD	E746-A750 del	10.473 ± 2.164
NCI-H1993	AD	WT	16.432 ± 0.965
NCI-H2126	LC	WT	23.179 ± 2.843
NCI-H1975	AD	L858R	26.748 ± 3.386
NCI-H1299	LC	WT	28.637 ± 2.512
NCI-H1648	AD	WT	38.425 ± 1.838
NCI-H1703	SQ	WT	41.258 ± 5.217
NCI-H2347	AD	WT	62.417 ± 4.658

*NSCLC* non-small cell lung carcinoma, *AD* adenocarcinoma, *SQ* squamous cell carcinoma, *LC* large cell carcinoma, *WT* wild type

### Potential regulation of miR-762 expression by the pro-inflammatory cytokine IL-6

**Fig. 2:**
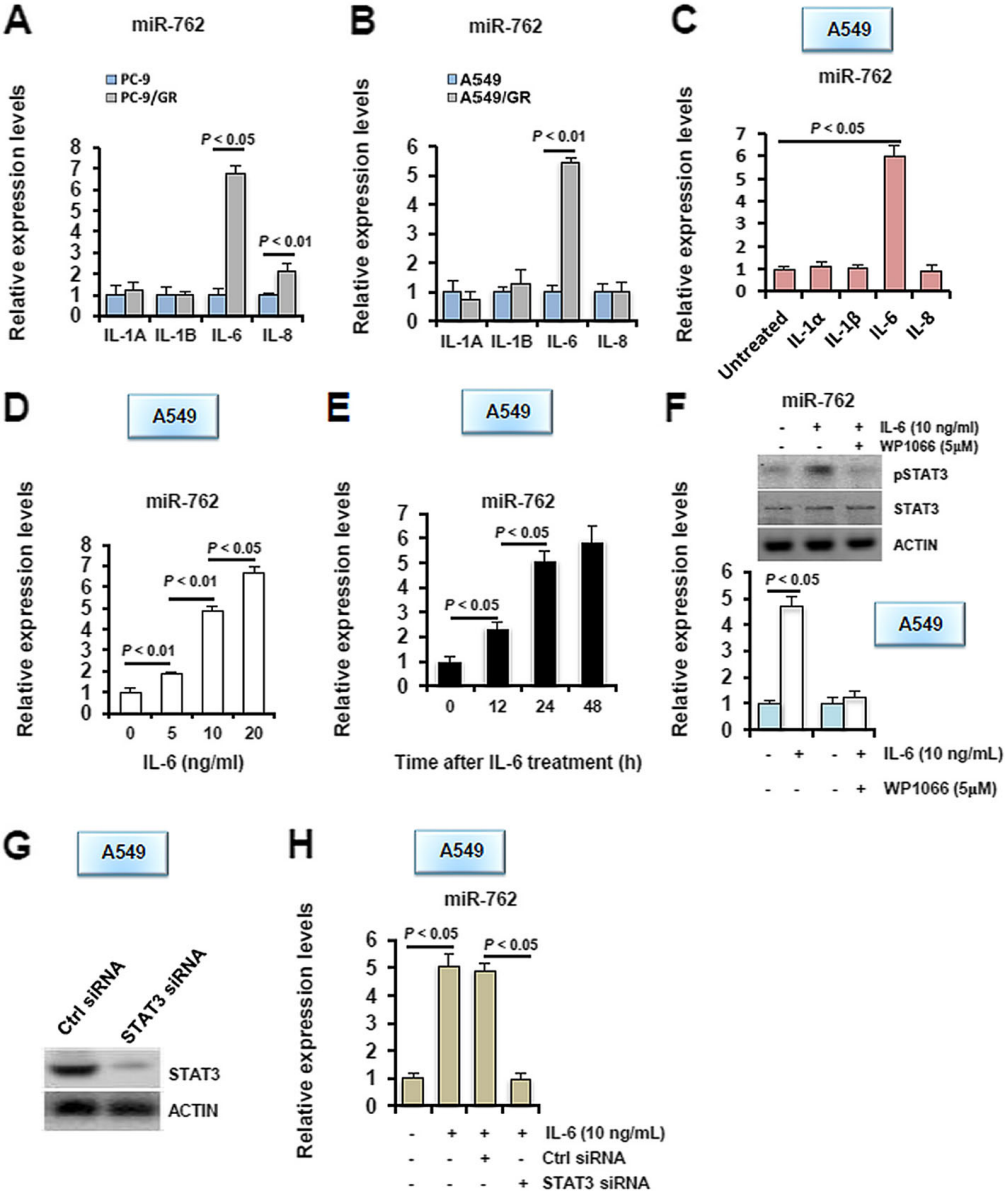
IL-6 serves as a potential upstream regulator of miR-762 induction in NSCLC cells. **a**-**b** Characterization of expression levels of different cytokines in different NSCLC cells using RT-qPCR. Each value is a mean ± S.E.M. from three independent experiments. **c** A549 cells were incubated with different cytokines, including IL-1α (5 ng/ml), IL-1β (10 ng/ml), IL-6 (10 ng/ml) and IL-8 (50 ng/ml) for 24 h, followed by RT-qPCR analysis of miR-762 expression. **d** A549 cells were stimulated with different doses of IL-6 for 24 h, followed by RT-qPCR analysis of miR-762 expression. **e** A549 cells were stimulated with 10 ng/ml of IL-6 for different durations as indicated, followed by RT-qPCR analysis of miR-762 expression. **f** A549 cells were stimulated with 10 ng/ml of IL-6, in the presence or absence or co-treatment with 5 μM of WP1066, for 24 h, followed by RT-qPCR analysis of miR-762 expression. Inhibition of STAT3 activation was verified using immunoblotting analysis of pSTAT3 expression (upper panel). **g** A549 cells were transiently transfected with *STAT3* siRNA or Ctrl siRNA. 48 h later, knockdown of STAT3 in A549 cells was validated using immunoblotting. **h** 48 h after siRNA treatment, A549 cells were stimulated with 10 ng/ml of IL-6 for 24 h, followed by RT-qPCR analysis of miR-762 expression

Because emerging data suggest that deregulated miR-762 expression is involved in immune regulation in different systems [20, 21], and because immune dysregulation represents one of the most important etiologies of gefitinib resistance in NSCLC [22], we therefore hypothesize that miR-762 actions on sensitivity to gefitinib may be exerted via modulation of immune response. In our ongoing study, we unexpectedly found that among different pro-inflammatory cytokines tested, expression levels of *IL-6* transcripts were induced unanimously in both PC-9/GR and A549/GR cells, compared to their parental cells (Fig. 2a-b). To be noted, IL-8 expression levels were observed to be increased in PC-9/GR cells compared to its parental PC-9 cells, but displayed no differential expression pattern in A549/GR and A549 cells (Fig. 2a). Considering that IL-8 signaling has been shown to be fundamentally involved in resistance of lung carcinoma cells to the EGFR TKI erlotinib [23], our results suggest that IL-8 action on response to EGFR TKIs may be dependent on cell context or types of TKIs-treatments. Despite the essential roles of miRNAs in human physiology and diseases, transcriptional regulation of miRNAs is so far poorly understood. To this end, emerging data have shown that certain inflammatory cytokines can function as potent regulators of miRNA expression. These cytokines and miRNAs form into a delicate regulatory network consisting of both feed forward and feedback loops, thus contributing crucially to cancerous progression [24]. The available data thus raise the possibility that miR-762 expression may be regulated by IL-6 signaling. Indeed, upon being challenged for 24 h with different recombinant human cytokines including IL-1α, IL-1β, IL-6 and IL-8, only IL-6 stimulated a significant elevation in miR-762 expression in A549 cells (Fig. 2c). This stimulatory effect was later found to be exerted in a dosage-dependent (Fig. 2d) and time-dependent (Fig. 2e) manner. To further determine whether the regulation of miR-762 expression by IL-6 was mediated through STAT3 signaling, we treated A549 cells for 24 h with 10 ng/ml of IL-6, in the presence or absence of the STAT3 inhibitor WP1066. Co-treatment with WP1066 concomitantly abolished IL-6-elicited pSTAT3 induction and miR-762 upregulation in A549 cells (Fig. 2f). To further validate this observation, we transiently knocked down the expression of endogenous STAT3 in A549 cells (Fig. 2), and then subjected these cells to IL-6 stimulation. Similarly as being treated by WP1066, ablation of STAT3 in A549 cells completely eliminated IL-6-induced miR-762 elevation (Fig. 2h). Moreover, the potential regulation of miR-762 by the IL-6/STAT3 cascade has been also verified in PC-9 cells (Additional file 1: Figure S1). We have thus identified a potential regulation of miR-762 by the IL-6/STAT3 cascade in NSCLC cells.

### miR-762 regulates sensitivity to gefitinib in NSCLC cells

**Fig. 3:**
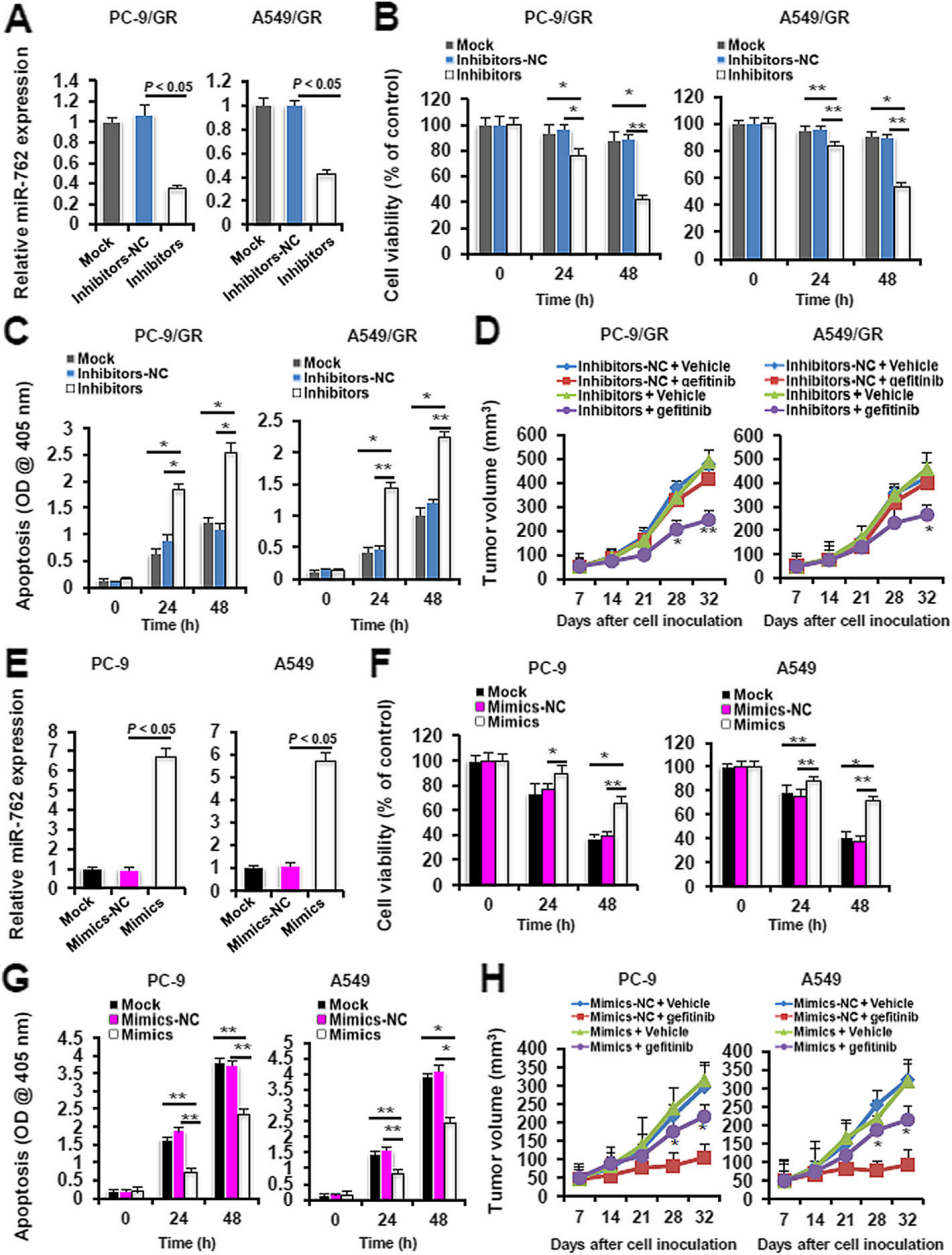
miR-762 upregulation desensitizes NSCLC cells to gefitinib treatment. **a** 48 h after transfection with miR-762 inhibitors or negative controls (NC), PC-9/GR and A549/GR cells were subjected to RT-qPCR analysis of miR-762 expression. **b** PC-9/GR and A549/GR cells were treated with different doses of gefitinib (8 μM for PC-9/GR, 60 μM for A549/GR) for 24 or 48 h. Cell viability was assayed using a MTT Assay Kit at 590 nm (*P < 0.05 and **P < 0.01). **c** Cells were treated with different doses of gefitinib (8 μM for PC-9/GR, 60 μM for A549/GR) for 24 or 48 h. Cell apoptosis was assayed using an ApoStrand™ ELISA Apoptosis Detection Kit at 405 nm (*P < 0.05 and **P < 0.01). **d** In vivo gefitinib sensitivity was evaluated using a xenograft model, as described in Materials and methods. Tumor volume was measured and recorded once a week (*P < 0.05 and ** *P* < 0.01 when comparing Inhibitors + vehicle to Inhibitors + gefitinib). **e** 48 h after transfection with miR-762 mimics or Mimics-NC, PC-9 and A549 cells were subjected to RT-qPCR analysis of miR-762 expression. **f** PC-9 and A549 cells were treated with different doses of gefitinib (0.2 μM for PC-9 and 12.5 μM for A549 cells) for 24 or 48 h. Cell viability was assayed using a MTT Assay Kit at 590 nm (*P < 0.05 and **P < 0.01). **g** PC-9 and A549 cells were treated with different doses of gefitinib (0.2 μM for PC-9 and 12.5 μM for A549 cells) for 24 or 48 h. Cell apoptosis was assayed using an ApoStrand™ ELISA Apoptosis Detection Kit at 405 nm (*P < 0.05 and **P < 0.01). **h** In vivo gefitinib sensitivity was evaluated using a xenograft model, as described in Materials and methods. Tumor volume was measured and recorded once a week (*P < 0.05 and **P < 0.01 when comparing Mimics + vehicle to Mimics + gefitinib)

Having established the association between miR-762 upregulation and impaired gefitinib chemosensitivity, we next explored the role of miR-762 in the modulation of gefitinib resistance by performing gain- and loss-of-function experiments. Transient transfection with miR-762 inhibitors caused a 67.6% and a 58.1% reduction in the miR-762 levels in PC-9/GR and A549/GR cells relative to transfection with inhibitors negative control (inhibitors-NC), respectively (Fig. 3a). Ablation of endogenous miR-762 in PC-9/GR and A549/GR cells significantly impaired cell viability (Fig. 3b) and potentiated apoptosis (Fig. 3c) at 24 to 48 h following gefitinib exposure. In accordance with these in vitro results, miR-762 inhibition in PC-9/GR and A549/GR cells noticeably suppressed tumor formation in the gefitinib-challenged xenograft model (Fig. 3d). To directly ask whether miR-762 could promote gefitinib resistance, we transiently overexpressed miR-762 mimics in PC-9 and A549 cells (Fig. 3e). As expected, augmentation of miR-762 expression in PC-9 and A549 cells dramatically enhanced cell viability (Fig. 3f) and attenuated apoptosis (Fig. 3g) at 24 to 48 h following gefitinib exposure. Likewise, overexpression of the exogenous miR-762 notably promoted tumor formation in the gefitinib-challenged xenograft model (Fig. 3h). These data collectively suggest that miR-762 could regulate sensitivity to gefitinib at both in vitro and in vivo levels in NSCLC cells.

### miR-762 directly targets ABR signaling in NSCLC cells

**Fig. 4:**
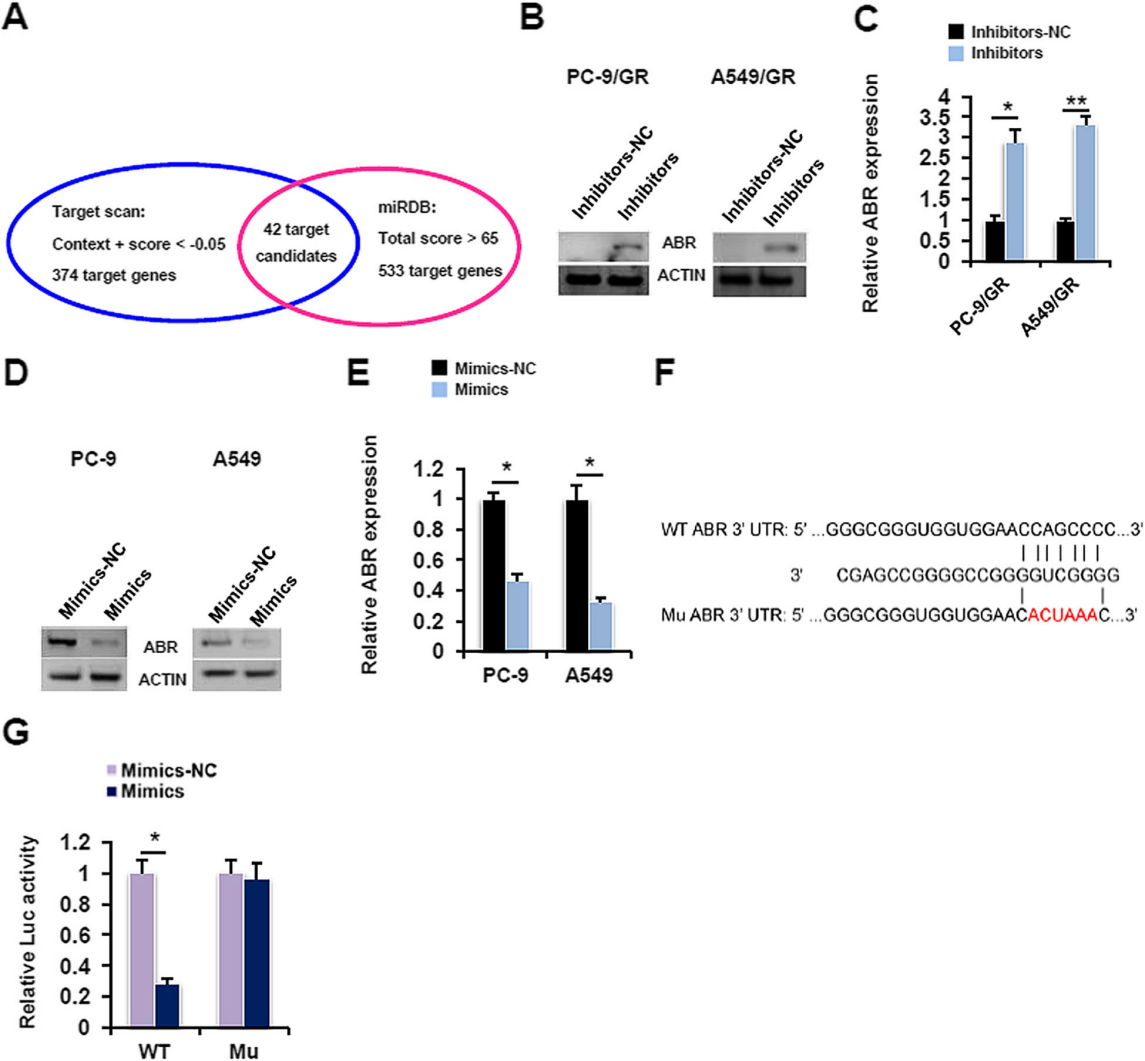
ABR serves as the direct target of miR-762 in NSCLC cells. **a** Prediction of putative target genes of miR-762 by Target scan and miRDB programs. **b** PC-9/GR and A549/GR cells were transfected with miR-762 inhibitors or inhibitors-NC for 48 h, followed by immunoblotting analysis of ABR expression. **c** PC-9/GR and A549/GR cells were transfected with miR-762 inhibitors or inhibitors-NC for 48 h, followed by RT-qPCR analysis of *ABR* expression (*P < 0.05 and **P < 0.01). **d** PC-9 and A549 cells were transfected with miR-762 mimics or mimics-NC for 48 h, followed by immunoblotting analysis of ABR expression. **e** PC-9 and A549 cells were transfected with miR-762 mimics or mimics-NC for 48 h, followed by RT-qPCR analysis of *ABR* expression (*P < 0.05 and **P < 0.01). **f** Predicted miR-762 binding sites in the 3′-UTR of *ABR* gene. **g** miR-762 mimics/Mimics-NC (25 pmol/well) and pGL3-ABR 3’UTR-Luc reporter (0.25 μg/well), together with 0.001 μg of the Renilla luciferase reporter (Promega, Beijing, China), were co-transfected into subconfluent proliferating NIH/3 T3 cells for 24 h, followed by measurement of the relative luciferase activity (*P < 0.05)

To dissect the molecular mechanisms underlying the aforementioned phenotype, we searched two public databases, namely Target scan and miRDB. 42 candidate genes were found to be potential targets of miR-762 by both programs (Fig. 4a, Additional file 5: Table S2). Among these candidate genes, we further chose to focus on active BCR related protein (ABR), mainly based on three reasons: i) We searched for potential targets of miR-762 based on those genes with oncogenic or apoptosis-regulating properties. To this end, ABR has been validated to be a potent tumor suppressor that plays an important role in chronic myeloid leukemia and meningiomas [25]. ii) Our in silico analysis revealed that 3′-UTR of human ABR contains a potential miR-762-binding site (data not shown). iii) Our preliminary expression profile analysis revealed that levels of miR-762 expression were negatively correlated to *ABR* mRNA levels in a 59-patient cohort (see below). The expression levels of miR-762 and endogenous *ABR* mRNA appeared to be negatively correlated, as shown in Fig. 1d-e and Additional file 2: Figure S2. Importantly, transient expression of miR-762 inhibitors in PC-9/GR and A549/GR cells significantly evoked the ABR expression at both protein (Fig. 4b) and mRNA (Fig. 4c) levels. In good contrast, upregulation of miR-762 expression by transient overexpression of miR-762 mimics effectively repressed the ABR expression in gefitinib-sensitive PC-9 and A549 cells (Fig. 4d and e). To directly assay the transcriptional regulation of *ABR* expression by miR-762, we employed a luciferase reporter assay. Co-transfection with miR-762 mimics and WT-pGL3-ABR 3’UTR-Luc reporter plasmids for 24 h in subconfluent proliferating NIH/3 T3 cells resulted in a 71.4% reduction in the *ABR* mRNA levels. This inhibitory effect was totally abolished when WT-pGL3-ABR 3’UTR-Luc reporter plasmids were replaced by Mu-pGL3-ABR 3’UTR-Luc reporter plasmids in the luciferase reporter assay (Fig. 4f). miR-762 thus represses the ABR expression by decreasing its mRNA stability in NSCLC cells.

### Ectopic expression of the exogenous ABR ameliorates miR-762-impaired gefitinib sensitivity in NSCLC cells

**Fig. 5:**
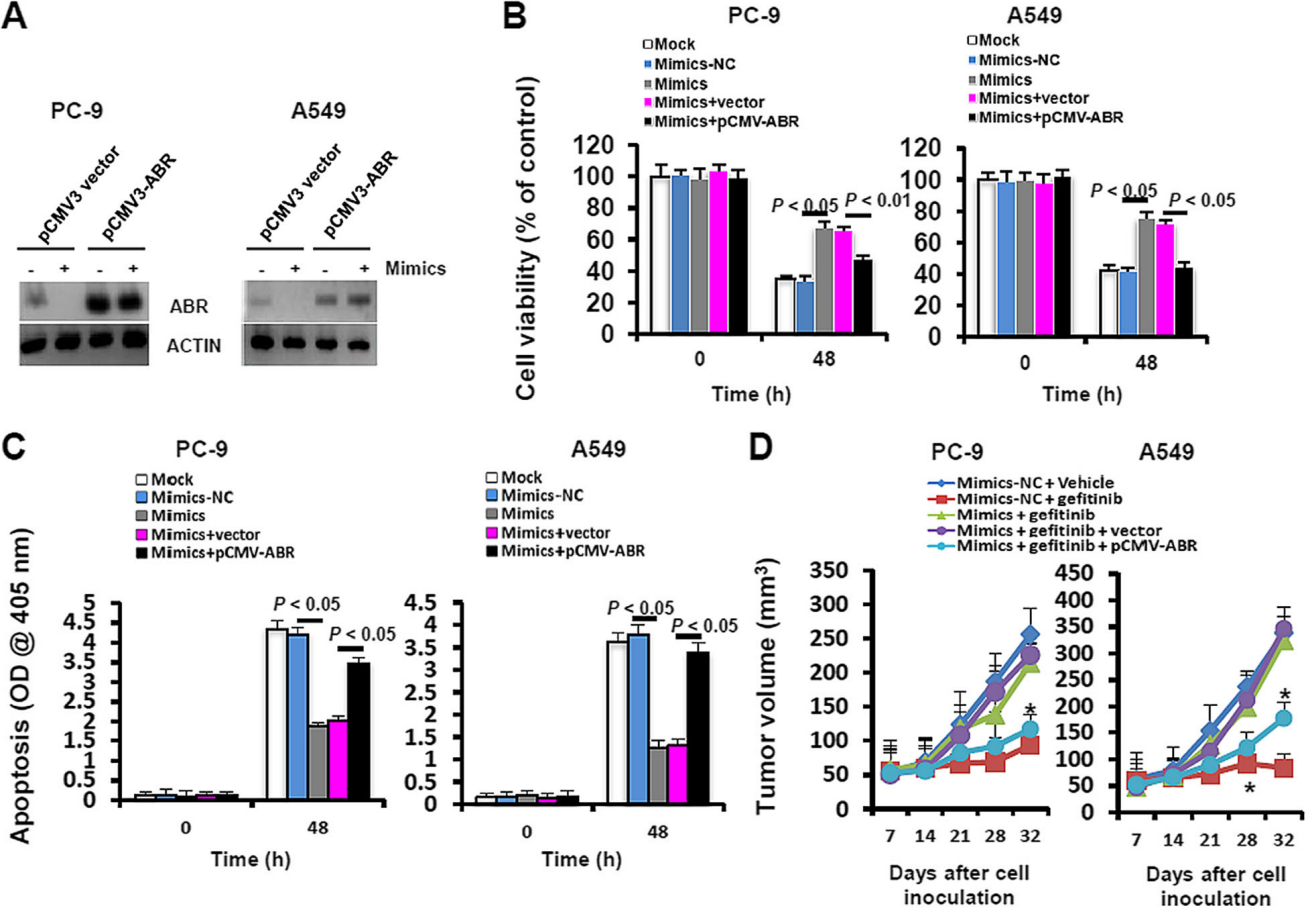
ABR overexpression alleviates miR-762-induced gefitinib resistance. **a** PC-9 and A549 cells that stably expressed the exogenous ABR was established as described in Materials and methods. PC-9/ABR and A549/ABR cells were transiently transfected with miR-762 mimics or mimics-NC for 48 h, followed by immunoblotting analysis of ABR expression. **b** 48 h after transfection with miR-762 mimics or Mimics-NC, PC-9/ABR and A549/ABR cells were treated with different doses of gefitinib (0.2 μM for PC-9/ABR and 12.5 μM for A549/ABR cells) for 48 h. Cell viability was assayed using a MTT Assay Kit at 590 nm. **c** 48 h after transfection with miR-762 mimics or Mimics-NC, PC-9/ABR and A549/ABR cells were treated with different doses of gefitinib (0.2 μM for PC-9/ABR and 12.5 μM for A549/ABR cells) for 48 h. Cell apoptosis was assayed using an ApoStrand™ ELISA Apoptosis Detection Kit at 405 nm. Different superscript letters denote groups that are statistically different (*P* < 0.05). **d** 48 h after transfection with miR-762 mimics or Mimics-NC, PC-9/ABR and A549/ABR cells were subjected to a xenograft model to measure the in vivo gefitinib sensitivity, as described in Materials and methods. Tumor volume was measured and recorded once a week (*P < 0.05 when comparing Mimics + gefitinib + vector to Mimics + gefitinib + pCMV3-ABR)

To further authenticate the involvement of ABR signaling in miR-762-impaired gefitinib sensitivity, we generated the PC-9 and A549 cells that stably expressed exogenous ABR. As shown by immunoblotting analyses, ABR expression was successfully restored in PC-9/ABR and A549/ABR cells, even in the presence of miR-762 mimics (Fig. 5a). Upon gefitinib challenge, transient transfection with miR-762 mimics caused a significant increase in cell viability in PC-9 and A549 cells, and this stimulatory effect was substantially abolished in PC-9/ABR and A549/ABR cells (Fig. 5b). Consistently, miR-762 mimics-repressed cell apoptosis in gefitinib-exposed LC cells was effectively restored by ABR overexpression (Fig. 5c). Moreover, transfection with miR-762 mimics in PC-9 and A549 cells notably promoted gefitinib resistance and increased tumor formation in a xenograft model. This stimulatory effect of miR-762 mimics was observed to be effectively reversed by ABR overexpression (Mimics + gefitinib + vector v.s. Mimics + gefitinib + pCMV-ABR, Fig. 5d). Thus, we have provided the evidence that the promoting effect of miR-762 mimics on gefitinib resistance in NSCLC cells is mediated mainly through the dysregulation of ABR pathway.

### Translational significance of deregulated miR-762 expression in EGFR-TKIs resistance in NSCLC

**Fig. 6:**
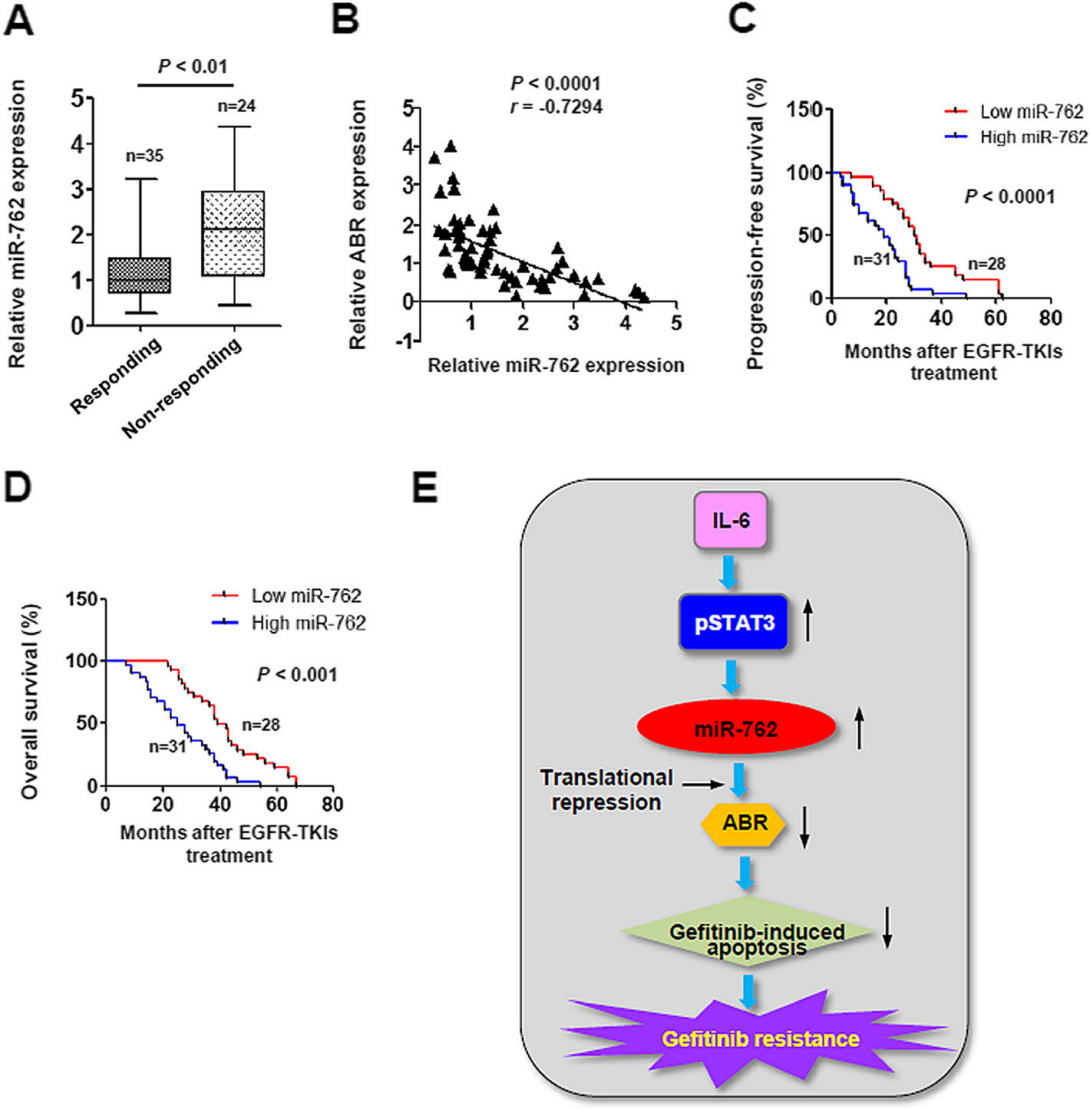
Translational significance of deregulated miR-762 expression in EGFR-TKIs-treated NSCLC. **a** RT-qPCR analysis of miR-762 expression in human EGFR-TKIs-responding, and EGFR-TKIs-non-responding NSCLC biopsies. **b** Expression levels of miR-762 and *ABR* mRNA in a total 59 of NSCLC tissue samples were determined using RT-qPCR analysis, followed by *Pearson Chi-Square* test. **c** Progression-free survival according to baseline miR-762 expression for 59 patients with EGFR-mutant NSCLC treated with first-line EGFR-TKIs. (**d**) (**c**) Overall survival according to baseline miR-762 expression for 59 patients with EGFR-mutant NSCLC treated with first-line EGFR-TKIs. **e** Proposed working model in the current study

We finally determined the clinical relevance of the current study by investigating the miR-762 expression status in a cohort of 59 patients with recurrent EGFR mutant NSCLC after the EGFR-TKIs therapy. miR-762 levels were significantly induced in EGFR-TKIs non-responding tissues relative to responding tissues (2.64 ± 0.97 v.s. 1.17 ± 0.84, *P* < 0.01, Fig. 6a). Furthermore, miR-762 expression was found to be negatively correlated to *ABR* mRNA levels in our cohort of 59 NSCLC patients who had previously received EGFR-TKIs treatment (*r* = − 0.7294, *P* < 0.0001, Fig. 6b). To further validate the clinical importance of miR-762 expression, we divided the subgroups (high/low miR-762) according to the median value as a cutoff [26]. Interestingly, high levels of miR-762 predisposed the NSCLC patients receiving EGFR-TKIs chemotherapy to a significantly shorter progression-free survival (Fig. 6c) and overall survival time (Fig. 6d). The available data thus point to a potent prognostic value of miR-762 in NSCLC patients after EGFR-TKIs therapy.

## Discussion

Recent microarray and RNA-sequencing studies have identified a bunch of miRNAs expressed aberrantly in gefitinib-resistant NSCLC cells [27]. The function and underlying mechanisms of these differentially expressed miRNAs, however, remain largely unknown. To this end, miR-762 was selected and subjected to further investigation based on three criteria: i) Misexpression of miR-762 during carcinogenesis and tumor progression has been reported previously by independent groups [6, 7, 28]. ii) Aberrant miR-762 expression has been shown in tumor samples and not just cell lines [28]. iii) Functional evidence of miR-762 as a conserved oncomiR in various tumor types has been presented by multiple publications [6, 7, 28]. We observed that miR-762 expression was significantly increased in experimentally established gefitinib-resistant NSCLC cells compared to the parental cells. A stepwise increase in miR-762 expression was even more prominent in different NSCLC cells with low or moderate sensitivity to gefitinib (Fig. 1), suggesting a close association between miR-762 dysregulation and pathogenesis of gefitinib resistance in NSCLC.

Despite much efforts focused on the identification of the down-stream targets of miRNAs, very little is known on the regulation of miRNAs themselves. To this end, our profiling assays have revealed that IL-6 may regulate miR-762 expression in NSCLC cells. This conclusion was drawn based upon three observations: i) IL-6 stimulated miR-762 expression in a dose- and time-dependent manner (Fig. 2c-e). ii) Blockage of the STAT3 activation using pharmacological or molecular biology approaches totally abrogated miR-762 upregulation in IL-6-challenged NSCLC cells (Fig. 2f-h). iii) Emerging data suggest that expression of miRNAs is primarily regulated at the level of promoter transcription [29]. Interestingly, with the aid of the PROmiRNA database, we have identified a putative STAT3 binding site in the 5′-UTR of pre-miRNA of hsa-miR-762 (Additional file 3: Figure S3). Based on the available data, we conclude that miR-762 expression may be regulated fundamentally by IL-6/STAT3 cascade in NSCLC cells. Previous studies have shown that IL-6/STAT3 activation could attenuate sensitivity to EGFR-mutant NSCLC cells to EGFR-TKIs including icotinib [30] and gefitinib [31]. Our findings extend these understandings by identifying miR-762 as a potential downstream effector of IL-6/STAT3 pathway in EGFR-TKIs-resistant NSCLC cells. This intriguing possibility is currently being given a full investigation in our lab.

In the context of malignant behavior, miR-762 expression has been shown to be notably upregulated in muscle-invasive bladder cancer [6] and ovarian cancer [7], and this upregulation frequently predicts a poor prognosis of corresponding patients. In agreement with these findings, our gain- and loss-of-function approaches demonstrates that elevated miR-762 enhances multiple aspects of malignant phenotypes including cell proliferation, resistance to gefitinib-induced apoptosis, thus potentiating cancerous progression in NSCLC (Fig. 3). The available data indicate that the oncogenic function may be an intrinsic character of miR-762. Given that aberrant miR-762 expression can also be found in plasma of patients with muscle-invasive bladder cancer [6] and Graves’ disease [32], future endeavor in this filed may help to screen an early, more convenient biomarker with potential diagnostic value.

By profiling NSCLC cells in the setting of ectopic miR-762 expression, we have identified ABR as the potential downstream target, and we have confirmed the direct binding of miR-762 to the 3′-UTR of *ABR* using multiple approaches including transient transfection, luciferase reporter assay and site-directed mutagenesis (Fig. 4). *ABR*, a homologue of *BCR* (breakpoint cluster region) gene, regulate a diversity of biological functions through negative modulation of the activation of the small GTPase Rac. Regarding the function/regulation of ABR, two fundamental aspects are worthy of note: i) ABR serves as a relatively conserved tumor suppressor in various solid tumors including medulloblastoma [33], breast cancer [34] and astrocytoma [35]. Nevertheless, whether ABR is subjected to transcriptional or posttranscriptional regulation remain unexplored. ii) Dysregulation of ABR function is associated with IL-6 activation. For example, in the absence of active ABR, hypoxia induces GTP-bound form of Rac, thus causing enhanced production of IL-6 during the pathogenesis of pulmonary hypertension [36]. The data shown here indicate that miR-762 is required for ABR inhibition by IL-6 in NSCLC cells. It will be of future interest to deconvolute a miR-762/ABR/Rac axis is also at play in EGFR-TKIs-resistant NSCLC.

The importance of the miR-762/ABR signaling in EGFR-TKIs resistance was finally validated in clinical samples from EGFR-mutant NSCLC patients. miR-762 levels were found to be significantly increased in EGFR-TKIs non-responding tissues relative to responding tissues. More importantly, high levels of miR-762 predisposed the NSCLC patients receiving EGFR-TKIs chemotherapy to a significantly shorter progression-free survival and overall survival time (Fig. 6). Of note, some patients harbor higher levels of miR-762 expression, and they also respond to gefitinib treatment properly (Fig. 6a). This is probably due to the fact that NSCLC is a heterogeneous set of cancer [37]. Anyway, our clinical data are based on a small size of NSCLC patients, and therefore have a certain limitation. A further study consisting of a larger scale of NSCLC patients is warranted thereof. Nevertheless, our clinical findings provide the first evidence for the potential usage of evaluating miR-762/ABR ratios as a molecular predictor of chemosensitivity to EGFR-TKIs in NSCLC.

## Conclusions

The data presented here have demonstrated that miR-762 upregulation, which is regulated, at least in part, by IL6/STAT3 signaling pathway, confers acquired resistance to gefitinib in NSCLC cells (Fig. 6e). From a clinical standpoint, miR-762 upregulation is tightly associated with the pathogenesis of gefitinib resistance, and may serve as a potent predictor of a poor response to EGFR-TKIs in NSCLC patients. In this context, miR-762 inhibition using systemic delivery (e.g. nanoparticles) may represent an attractive therapeutic strategy for patents with recurrent EGFR mutant NSCLC.

## Supplementary information

**Additional file 1: Figure S1.** Verification of the regulation of miR-762 expression by IL-6 signaling pathway in PC-9 cells. (A) PC-9 cells were incubated with different cytokines, including IL-1α (5 ng/ml), IL-1β (10 ng/ml), IL-6 (10 ng/ml) and IL-8 (50 ng/ml) for 24 h, followed by RT-qPCR analysis of miR-762 expression. (B) PC-9 cells were stimulated with different doses of IL-6 for 24 h, followed by RT-qPCR analysis of miR-762 expression. (C) PC-9 cells were stimulated with 10 ng/ml of IL-6 for different durations as indicated, followed by RT-qPCR analysis of miR-762 expression. (D) PC-9 cells were transiently transfected with *STAT3* siRNA or Ctrl siRNA. 48 h later, knockdown of STAT3 in A549 cells was validated using immunoblotting. (H) 48 h after siRNA treatment, PC-9 cells were stimulated with 10 ng/ml of IL-6 for 24 h, followed by RT-qPCR analysis of miR-762 expression.

**Additional file 2: Figure S2.** Characterization of *ABR* mRNA expression in different NSCLC cells using RT-qPCR. The value indicates the relative expression levels of *ABR* mRNA in the cells (PC-9/PC-9/GR and A549/A549/GR cells) at different batches of gefitinib resistant induction.

**Additional file 3: Figure S3.** Identification of a putative STAT3 binding site in the 5′-UTR of pre-miRNA of hsa-miR-762 using the PROmiRNA database.

**Additional file 4: Table S1.** Details of antibodies used in the current study.

**Additional file 5: Table S2.** 42 candidate genes of miR-762 predicted by Target scan and miRDB programs in the current study.

## Data Availability

All data generated or analyzed during this study are included in this published article [and its supplementary information files].

## Abbreviations

ABR: Active BCR related protein
ANOVA: Analysis of variance
ATCC: American Type Culture Collection
EGFR: Epidermal growth factor receptor
ELISA: Enzyme-linked immunosorbent assay
IL-6: Interleukin-6
LC: Lung cancer
MTT: Methyl thiazolyl tetrazolium
NSCLC: Non-small-cell lung cancer
qPCR: Quantitative polymerase chain reaction
RT: Reverse transcription
SDS-PAGE: Sodium dodecyl sulphate-polyacrylamide gel electrophoresis
TKIs: Tyrosine kinase inhibitors
