# Supplementary figures and images for: miR-762 activation confers acquired resistance to gefitinib in non-small cell lung cancer
Source: BMC Cancer. 2019 Dec 10;19:1203. doi: 10.1186/s12885-019-6416-4 (PMC6905032; doi:10.1186/s12885-019-6416-4)

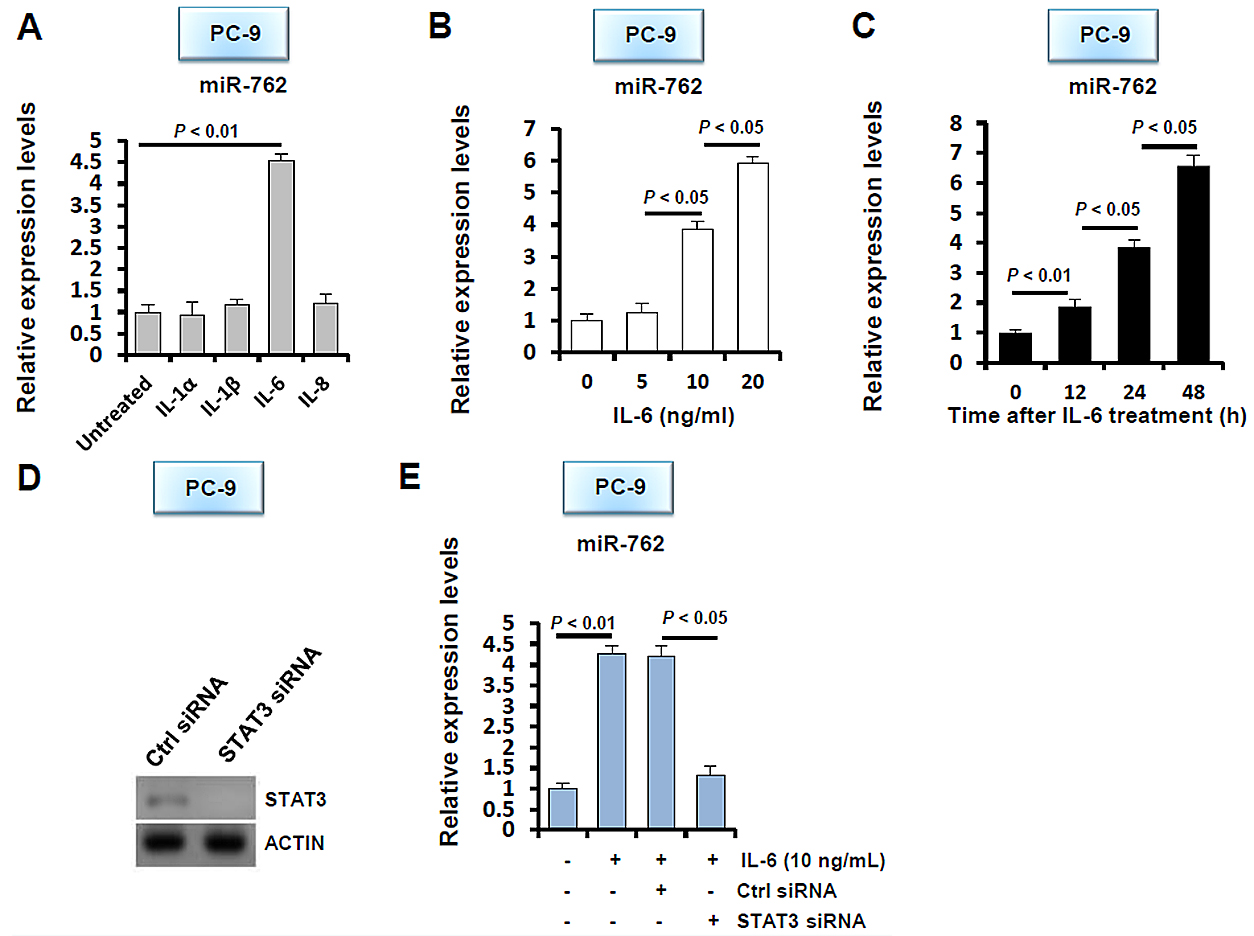

Supplement: Supplementary file 1 — Additional file 1: Figure S1. Verification of the regulation of miR-762 expression by IL-6 signaling pathway in PC-9 cells. (A) PC-9 cells were incubated with different cytokines, including IL-1α (5 ng/ml), IL-1β (10 ng/ml), IL-6 (10 ng/ml) and IL-8 (50 ng/ml) for 24 h, followed by RT-qPCR analysis of miR-762 expression. (B) PC-9 cells were stimulated with different doses of IL-6 for 24 h, followed by RT-qPCR analysis of miR-762 expression. (C) PC-9 cells were stimulated with 10 ng/ml of IL-6 for different durations as indicated, followed by RT-qPCR analysis of miR-762 expression. (D) PC-9 cells were transiently transfected with STAT3 siRNA or Ctrl siRNA. 48 h later, knockdown of STAT3 in A549 cells was validated using immunoblotting. (H) 48 h after siRNA treatment, PC-9 cells were stimulated with 10 ng/ml of IL-6 for 24 h, followed by RT-qPCR analysis of miR-762 expression. [file 12885_2019_6416_MOESM1_ESM.jpg]

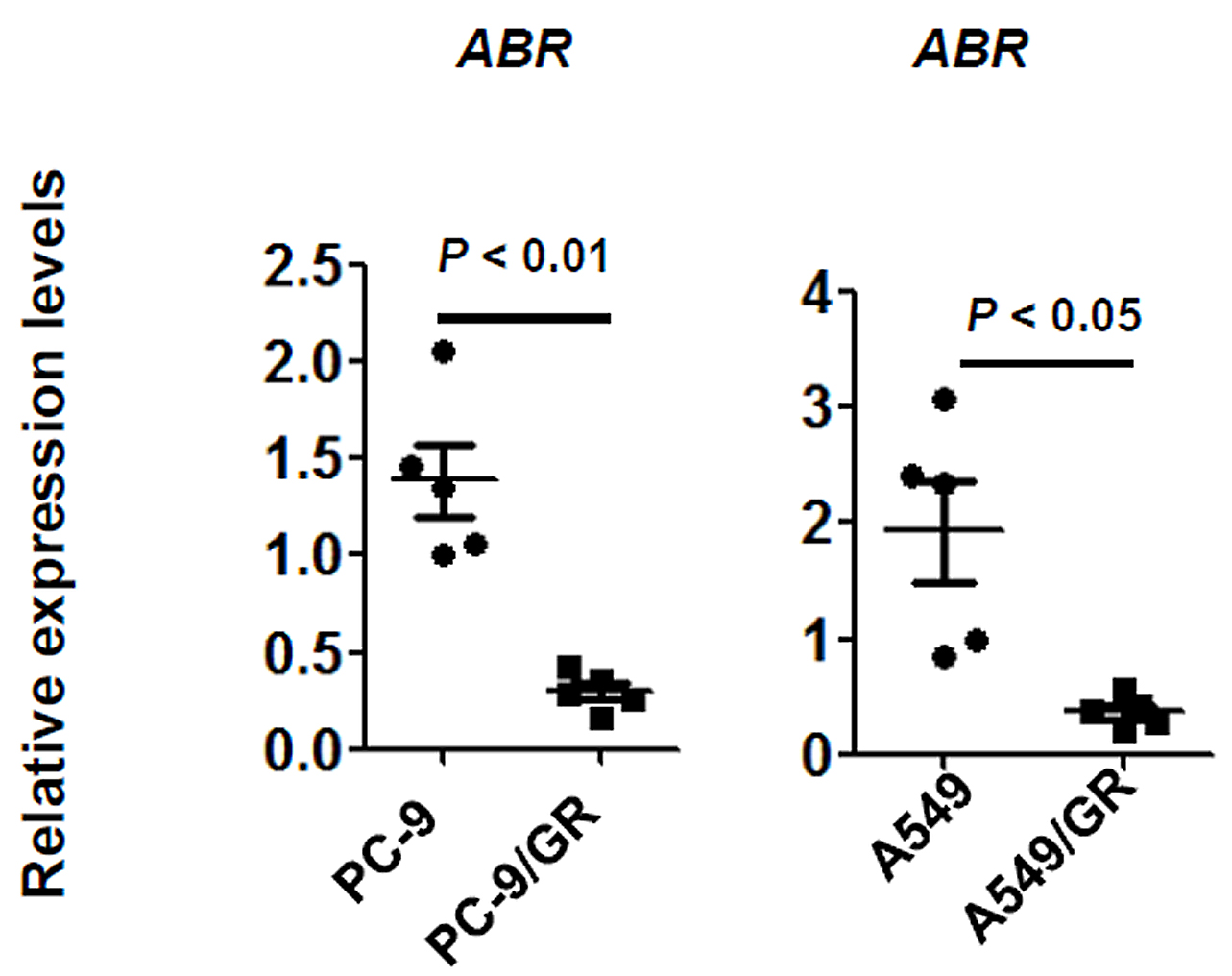

Supplement: Supplementary file 2 — Additional file 2: Figure S2. Characterization of ABR mRNA expression in different NSCLC cells using RT-qPCR. The value indicates the relative expression levels of ABR mRNA in the cells (PC-9/PC-9/GR and A549/A549/GR cells) at different batches of gefitinib resistant induction. [file 12885_2019_6416_MOESM2_ESM.jpg]

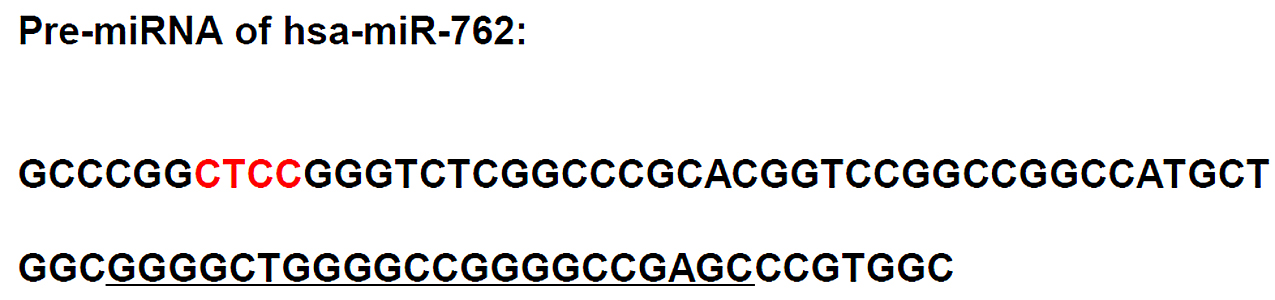

Supplement: Supplementary file 3 — Additional file 3: Figure S3. Identification of a putative STAT3 binding site in the 5′-UTR of pre-miRNA of hsa-miR-762 using the PROmiRNA database. [file 12885_2019_6416_MOESM3_ESM.jpg]
