# Supplementary material for: miR-762 activation confers acquired resistance to gefitinib in non-small cell lung cancer
Source: BMC Cancer. 2019 Dec 10;19:1203. doi: 10.1186/s12885-019-6416-4 (PMC6905032; doi:10.1186/s12885-019-6416-4)
Supplement: Supplementary file 4 — Additional file 4: Table S1. Details of antibodies used in the current study. [file 12885_2019_6416_MOESM4_ESM.doc]

Additional file 4: **Table S1** Details of antibodies used in the current study

| **Name** | **Company** | **Application (IB)** |
| --- | --- | --- |
| Rabbit anti-pSTAT3 | Abcam | 1:1000 |
| Rabbit anti-STAT3 | Abcam | 1:2000 |
| Rabbit anti-ACTIN | Sigma-Aldrich | 1:5000 |
| Rabbit anti-ABR | Abcam | 1:1000 |
| Goat anti-Rabbit 2^nd^ Antibody-HRP | Thermo Fisher Scientific | 1:10000 |
